# Norbornene as Key for a Possible Efficient Chemical Recycling in Structures Based on Ethylene

**DOI:** 10.3390/polym14225052

**Published:** 2022-11-21

**Authors:** Antonio F. Calles-Valero, Alberto García-Peñas, María L. Cerrada, José M. Gómez-Elvira

**Affiliations:** 1 Departamento de Ciencia e Ingeniería de Materiales e Ingeniería Química, IAAB, Universidad Carlos III de Madrid, Avda. de la Universidad, 30, 28911 Leganés, Spain; 2 Instituto de Ciencia y Tecnología de Polímeros (ICTP-CSIC), Juan de la Cierva 3, 28006 Madrid, Spain

**Keywords:** degradation, polyolefins, polyethylene, norbornene, chemical recycling

## Abstract

Different circular strategies attempt to increase the energy efficiency or reduce the accumulation of plastic in landfills through the development of circular polymers. Chemical recycling is essential to recover the initial monomers from plastic residues for obtaining new goods showing the same properties as those using virgin monomers from the initial feedstocks. This work addresses the preparation of poly(ethylene-*co*-norbornene) copolymers for a promising generation of materials for energy applications that could be treated by chemical recycling. The thermal and thermo-oxidative stability for these copolymers with norbornene is higher than for the neat PE, while their degradation exhibits an activation energy lower than that observed in PE, pointing out that chemical recycling would require a lower energy consumption.

## 1. Introduction

New materials with specific properties in terms of energy storage are required for the industry to reach the goal proposed through the 2050 long-term strategy, as an essential part of the European Green Deal and the Paris Agreement. Those new materials should accomplish circularity aspects, allowing reduction of the accumulation of residues derived from electronic devices in landfills.

Chemical recycling is a very promising route forward in the production of circular polymers, and an effective solution to the negative impact of plastics in the environment. The interest in such technology arises from the fact that it allows obtaining high added-value chemicals, which could significantly contribute to the removal of plastic waste [1,2,3]. Accordingly, one of the current challenges in the polymer industry is to develop materials that are not only more advanced, but also more sustainable, being able to be reconverted at the end of their service life into valuable products or raw materials. Fuels are one of these, as their production from plastic residues would have the additional benefit of diminishing the consumption of non-renewable feedstocks. Polyolefin-rich residues are, in this case, priceless, as their aliphatic nature makes them suitable to be pyrolyzed into fuel-type alkanes [4,5,6].

Another important issue is the economic feasibility of chemical recovery, which depends mainly on its energy consumption. In this respect, polymeric chain microstructure is a key aspect to make chemical recycling attractive, together with the use of suitable catalysts to minimize the activation energy of pyrolysis [5,7,8,9,10]. Composition, molecular weight and tacticity have been found to strongly influence the activation energy of polypropylene (PP)-based polyolefins [11,12,13,14], and are thus worth considering when evaluating the circular character of these materials via pyrolysis conversion. Accordingly, new polyolefins should be engineered to fit requirements for special applications, avoiding their widespread presence in single-use items, without overlooking that their final pyrolytic conversion into low-molecular weight species must be viable from an economic point of view.

**Scheme 1:**
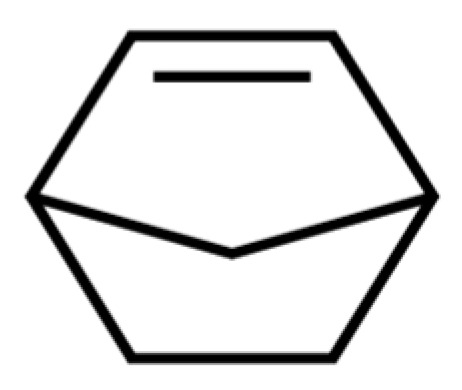
Chemical structure of norbornene.

One current use of polyolefins as specialties is as dielectrics in film capacitors, where isotactic PP is mainly preferred due to its crystallinity and low permittivity, which allow it to withstand high-voltage fields while keeping electric losses very low. Incorporation of cyclic olefin monomers, such as norbornene (see its chemical structure in Scheme 1), allows the synthesizing of the so-called cyclic olefin copolymers (COC), which show a more rigid amorphous phase, increasing mechanical stability and maintaining the dielectric permittivity as low at high operating temperatures.

The COC materials studied are mostly based on polyethylene (PE)-based ones; in fact, different industrial grades can be found in the market. They are amorphous, and thus exhibit high transparency and gloss. Their characteristic profiles can be varied over a wide interval by adjusting the chemical composition during synthesis; for instance, their glass transition temperature (T_g_) significantly increases as cyclic olefin content is raised [15,16,17,18]. Nevertheless, some efforts have been made toward studying their thermal stability under both oxidative and inert conditions [17,19], in order to find out whether their use is beneficial for high-temperature applications, such as in capacitor dielectrics, as well as to reduce energy consumption in potential pyrolytic recovery processes.

The present work aims the synthesis and microstructural characterization of a series of poly(ethylene-*co*-norbornene) materials in order to delve into the parameters that drive the changes detected in the PE thermal stability under air and N_2_ atmospheres. The purpose of this is to lay the basis for the use of COC copolymers as materials resistant against thermo-oxidation, either by themselves or as blending components, and to upgrade these commodities into high-performance materials. Furthermore, pyrolysis analysis intends to evaluate the contribution of such materials to make PE chemical recycling cost effective. The study is restricted to a rather narrow range in composition, to show how insertion of small norbornene contents leads to significant variations in ultimate properties, such as thermal stability, and can ultimately be related to new copolymer chain microstructures.

## 2. Experimental

### 2.1. Materials

Ethylene (99.95%, Nippon Gases, Madrid, Spain) and nitrogen (99.998%, Carburos Metálicos, Madrid, Spain) were purified through oxygen-trap columns and molecular sieves before use. Norbornene (99%, Aldrich, Madrid, Spain) and toluene (99.7–99.9%, Merck, Madrid, Spain) were refluxed over sodium (99%, Panreac, Barcelona, Spain), and distilled to avoid the presence of water traces and oxygen. Both were stored under a nitrogen atmosphere. The *rac*-dimethylsilylbis(1-indenyl) zirconium dichloride (Strem), modified methylaluminoxane (MMAO, 10 wt% in Aluminum, Molekula, München, Germany), ethanol (96%, Aroca, Valdemoro, Spain), Hydrochloric acid (HCl, 37%, VWR Chemicals, Barcelona, Spain), and 1,1,2,2-Tetrachloroethane-d_2_ (C_2_D_2_Cl_4_ ≥ 99.5 D, Aldrich) were used as received.

### 2.2. Synthesis of Polyethylene

**Scheme 2:**
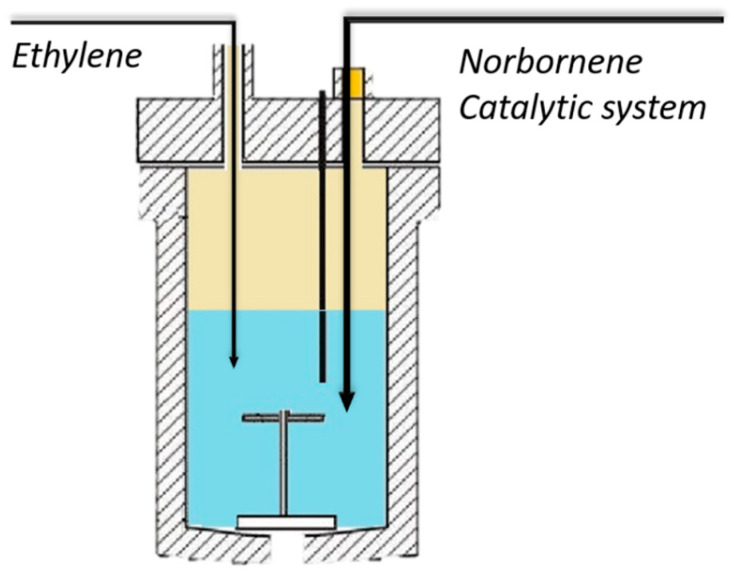
Autoclave reactor used for the synthesis.

The preparation of polyethylene was carried out in a Büchi glass Ecoclave (500 mL) using toluene as solvent (250 mL) at 10 °C, as shown in Scheme 2. The ethylene is added until a pressure of 1.58 bar is reached, and the catalytic system is subsequently added, which is composed by *rac*-dimethylsilylbis(1-indenyl) zirconium dichloride and MMAO ([Al]/[Zr] = 1082). The reaction is stopped at 20% of ethylene consumption by adding 5 mL of ethanol inside the reactor and purging the ethylene of the reactor. The homopolymer was precipitated in a mixture of ethanol and HCl (30:1). The powder was stirred and washed with ethanol overnight, filtrated, and then washed again with ethanol. Finally, the resulting polymer was dried under a vacuum at room temperature.

The ethylene concentration inside the reactor was estimated following the procedure reported elsewhere [20]. The expression used for this calculation relates the ethylene partial pressure to its concentration in toluene, as follows

(1) $${\left[E\right]}_{\mathrm{mol}/L}=P_{partial}\;\left(E\right)\cdot 12.998\cdot 10^{-5}\cdot e^{\frac{2215.7}{T}}$$

where *P_partial_ (E)* is the initial pressure of ethylene (atm) and *T* is the temperature (K) of the system.

### 2.3. Synthesis of Copolymers Based on Ethylene and Norbornene

The copolymers were prepared following the same procedure as described for the homopolymer, but the norbornene was injected into the reactor after the addition of the toluene. In a similar way, the reaction is stopped and final powder is treated.

Table 1 exhibits the polymerization conditions for all the samples prepared, where the initial pressure and the norbornene/ethylene ratios are shown for all the samples, including the homopolymer. The ratio between the co-catalyst and catalyst, expressed by the [Al]/[Zr] molar relationship, was kept constant. The copolymers are called cEN, followed by the content of norbornene, and the polyethylene is identified as PE.

### 2.4. Materials Processing

The polymers were processed by 2 min compression molding in a Collin 200 × 200 press between hot plates, at a temperature of 30 °C above their corresponding melting points, and using different pressures depending on the content of norbornene (Table 2). Slightly higher pressures and temperatures were required for cEN19. The subsequent cooling down to room temperature was performed between refrigerated plates under the same pressure, simulating an industrial processing.

### 2.5. Nuclear Magnetic Resonance (^13^C NMR)

The ^13^C NMR spectra of homopolymer and copolymers were obtained with a JEOL JNM-ECZ400R NMR spectrometer (JNM-ECZR series) from polymeric solutions prepared in 1,1,2,2-tetrachloroethane-*d_4_* (40 mg/1.2 mL) at 100 °C. The measurements were carried out at 100 MHz, using an acquisition time of 1 s, a relaxation delay of 5 s, a pulse angle of 45° and a minimum of 8000 scans.

### 2.6. Fourier Transform Infrared Spectroscopy

The FTIR spectra of the resulting materials were recorded from the films, by carrying out four scans in a Perkin Elmer Spectrum Two, in the 4000–450 cm^−1^ range, at a resolution of 2 cm^−1^. The attenuated total reflection mode (ATR-FTIR) was performed with a force gauge of 60. All the spectra were baseline corrected and normalized to the area of the 1500–1397 cm^−1^ region. The oxidation degree of samples was analyzed through the 1712 cm^−1^ peak (carbonyl index-C.I.), which is associated with saturated ketones and acids [21].

### 2.7. Differential Scanning Calorimetry

The glass transition temperature (T_g_) of the processed materials was analyzed in a DSC822e differential scanning calorimeter connected to a cooling system. The calorimeter was calibrated with indium and zinc. The film samples were heated from 0 °C to 225 °C, at a heating rate of 10 °C min^−1^. They were then subsequently cooled at 10 °C min^−1^ down to −60 °C, and finally heated again up to 225 °C. The values of T_g_ were estimated from those calorimetric curves after the second heating scan.

### 2.8. Thermogravimetry

The thermal stability of the samples was analyzed using a TGAQ 500 under inert and oxidative atmospheres. The samples used were small discs (around 5 mm diameter) obtained from the prepared films, whose weight was approximately 5 mg. Heating ramps from 25 °C up to 700 °C at 2, 5, 10 and 20 °C min^−1^ were applied, under a 90 mL min^−1^ flow of nitrogen or air.

### 2.9. Dynamic Mechanical Analysis (DMA)

Storage moduli of the PE homopolymer and the different COC copolymers were measured with a TA Q800 Dynamic Mechanical Thermal Analyzer, working in a tensile mode. The values were determined as function of temperature over the range from –150 to 125 °C at a fixed frequency of 3 Hz, and at a heating rate of 1.5 °C/min. For this analysis, strips of 2.2 mm width and 15 mm length were cut from the molded films.

## 3. Results and Discussion

### 3.1. Kinetics and Catalytic Activity

The polymers were synthesized following the procedure explained in the experimental part, obtaining relatively high yields in a fairly broad range of compositions. Table 3 shows the most relevant data of the reactions, among which the global activity (A) has been calculated from the mass of the resulting polymer (mass_polymer_), time of polymerization (t_reaction_) and the amount of catalyst (mol_catalyst_) used, according to expression (2):

(2) $$\;\;\;\;\;\;\;\;\;\;\;\;\;\;\;\;\;\;\;\;\;\;A=\frac{mass_{polymer}}{t_{reaction}\cdot mol_{catalyst}}$$

**Figure 1:**
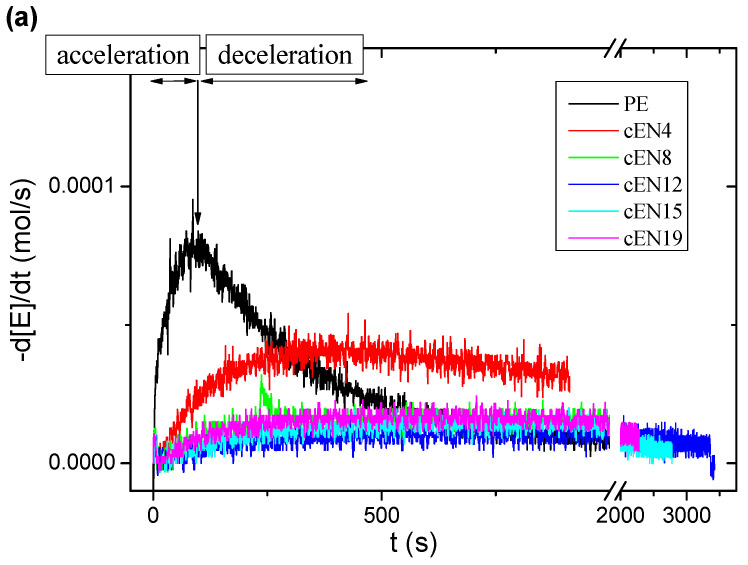

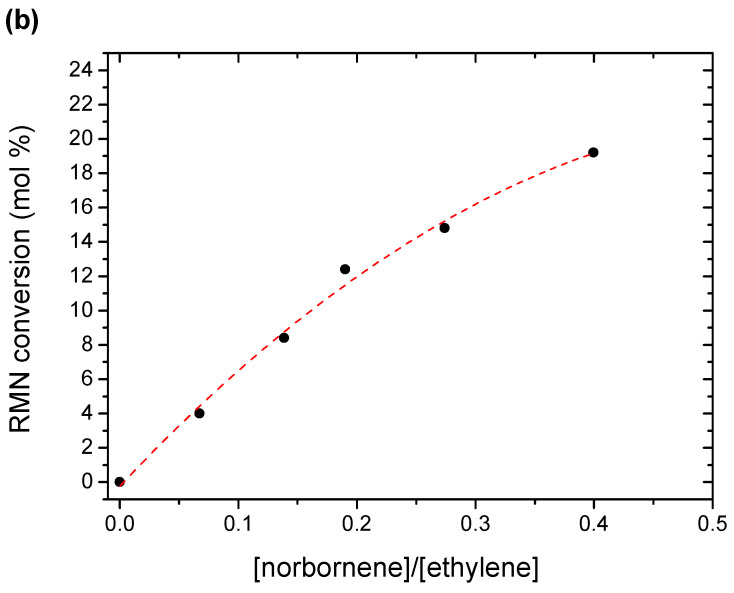
Polymerization kinetics for the homopolymer and copolymers (**a**), and molar % conversion regarding to the molar relationship between monomers (**b**).

The pressure drop with time was measured for all reactions. The corresponding derivative allows the obtaining of reaction rates, as represented in Figure 1a. The curves clearly exhibit two steps associated with acceleration and deceleration of the polymerization, but their shape strongly depends on the content of norbornene within the reaction medium.

A small amount of norbornene radically reduces the reaction rate regarding the preparation of homopolymer (PE). This tendency is sharpened if the norbornene content increases up to reach values around 8 mol %, but for higher contents, the shape is barely modified, i.e., similar kinetics curves are observed.

All the reaction rate curves reach a maximum (r_max_), whose intensity and associated time decrease with the norbornene content in the feeding. This effect in the kinetics is contrary to the one described for long chain α-olefins, where the presence of low contents of the comonomer speeds up the insertion kinetics. Such a negative effect could be explained in terms of the steric hindrance of norbornene units to be coordinated with the catalyst, and subsequently inserted into the polymer chain.

Despite the fact that the deceleration in the kinetics is already apparent in the cEN4 copolymerization, the global activity, as measured from the weight of copolymer obtained (Table 3), increases for this case. These seemingly contradictory facts can be brought into line by considering changes in the reaction medium while reaction is taking place. Indeed, the ethylene homopolymerization exhibits a sharp speeding up that causes the active centers to be quickly entrapped inside highly insoluble polymer chain clusters that hinder the monomer accessibility. This is reflected in a harsh deceleration in the ethylene consumption, and therefore in the decrease of polymer production. On the other hand, the insertion of a small content of norbornene increases the solubility of chains, which contributes to keep catalyst centers available for polymerization. It is also clear that the presence of higher norbornene contents causes the kinetics to be controlled by the slow insertion of the cyclic comonomer, thereby leading to a decrease in global activity that is consistent with the diminution of the reaction rate.

Similar to polymerization kinetics, the copolymer composition depends on the ratio of both comonomer units in the feeding. Figure 1b shows a linear relationship between the conversion and the norbornene content up to a feed molar relationship of 0.2, and clearly fits with a quadratic equation for higher feeding ratios.

### 3.2. Molecular Characterization

**Scheme 3:**
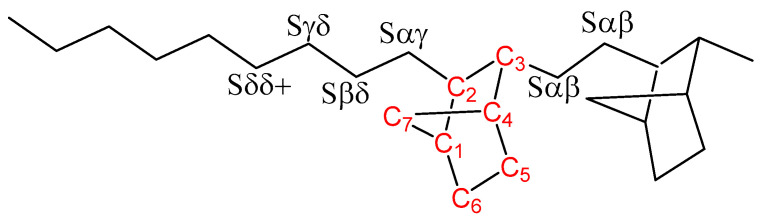
Identification of carbons associated with both ethylene (in black) and norbornene (red).

The whole norbornene content (mol %) within the polymeric chains is estimated by ^13^C NMR, from the signals corresponding to the different carbons, as was previously reported by Tritto et al. [16], by using Equation (3) indicated below [7]. This expression considers all carbon nuclei as identified in Scheme 3.

(3) $$X_{N}=\frac{\frac{1}{5}\cdot \left[I\left(C2,\;C3\right)+I\left(C1,\;C4\right)+I\left(C7\right)\right]}{\frac{1}{2}\cdot \left[I\left(C5,\;C6\right)+I\left(\mathrm{CH}2-\mathrm{ethylene}\right)\right]}=\frac{2}{5}\cdot \frac{5N}{2N+2E}=\frac{I\left(A\right)+I\left(B\right)+I\left(C\right)}{2.5\cdot I\left(D\right)}$$

**Figure 2:**
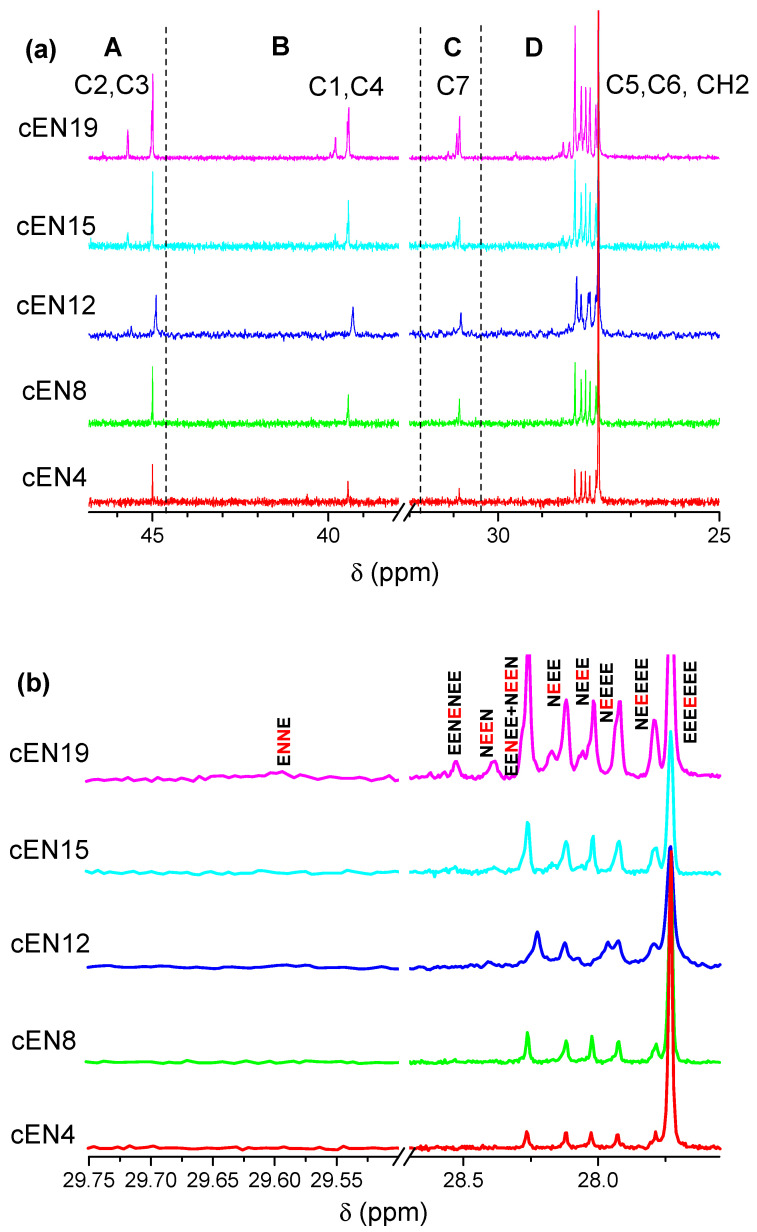
^13^C RMN spectra for the obtained copolymers (**a**), and identification of compositional sequences associated with ethylene (E) and norbornene (N) units (**b**).

In-chain methylene carbons are named according to their relative distances to the norbornene unit on both sides, and carbons belonging to the norbornene are numbered from C1 to C7. The corresponding signals are indicated in Figure 2a, as well as the four windows (from A to D) in which they can be grouped. In addition, Figure 2b shows the specific compositional sequences derived from the methylene carbons of ethylene units indicated in Scheme 1, the assignment of which can be seen in Table 4.

A higher amount of comonomer in the reaction medium promotes the diversity of sequences composed by ethylene and norbornene, as can be deduced from the increasing multiplicity of CH_2_ signals in Figure 2a (D region). The distribution of comonomers at the triad level is collected in Table 5, where the average ethylene length (*n_E_*), calculated from Expression (4) [22], is also shown. According to these data, the most relevant microstructural features are, firstly, that only isolated norbornene units are present, except for sample cEN19, which exhibits traces of *NN* diads; and secondly, that isolated ethylene diads (*NEEN*) appear from sample cEN12, while isolated E units are exclusive of samples cEN15 and cEN19. Finally, it is worth noting that alternating sequences (*ENEN*) are not detected, not even in the cEN19 sample.

(4) $$n_{E}=\frac{EEE+EEN+NEN}{NEN+\frac{1}{2}\cdot EEN}$$

**Figure 3:**
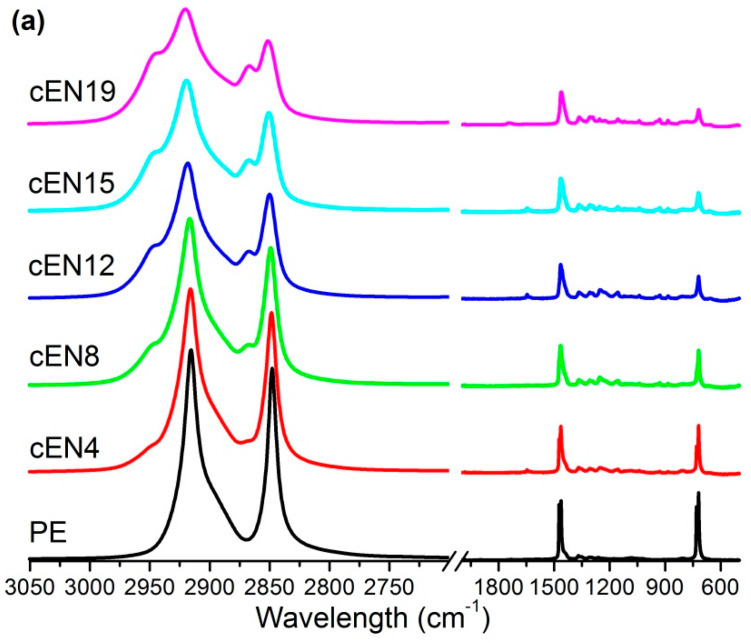

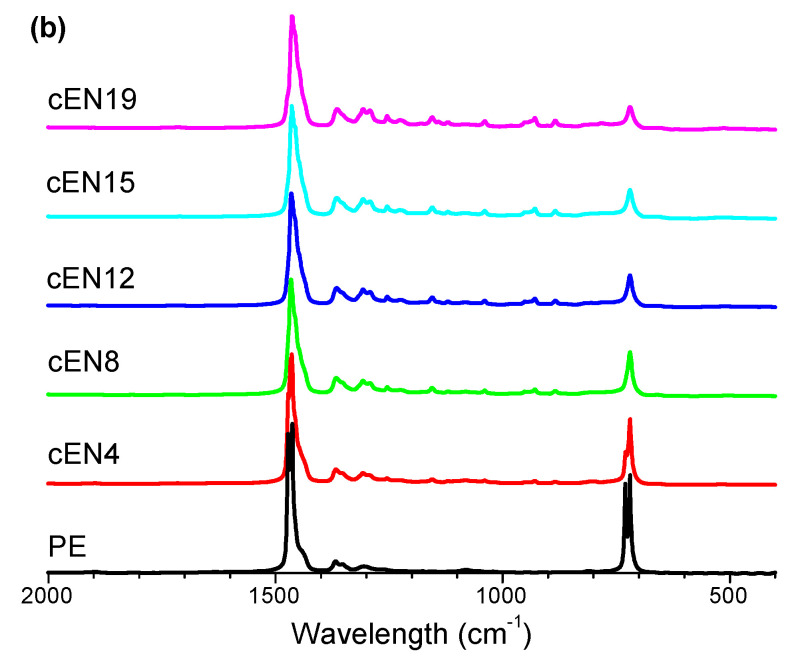
ATR-FTIR spectra for all the samples (**a**), and normalized spectra ranging between 2000 and 400 cm^−1^ (**b**).

The materials were also characterized by ATR-FTIR spectroscopy to obtain clean spectra without saturated bands, and then to detect distinctive absorptions of norbornene units in the whole wave number range. The spectra are shown in Figure 3, where it can be seen that the increase of norbornene content causes the bands placed at 2944 cm^−1^ and 2868 cm^−1^ to show up, and at the same time, the bands located at 2916 cm^−1^ and 2848 cm^−1^ to decrease (Figure 3b). However, despite the clear assignment of these vibration modes to C-H stretching of norbornene and ethylene units, respectively, the overlapping does not allow the estimation of the composition of copolymers.

As for the region covering the C-H deformation modes (Figure 3a), the area placed between 1005 and 840 cm^−1^ has been identified as distinctive and exclusive for norbornene, and therefore suitable to be taken as indicative of the norbornene content in FTIR analysis.

Another interesting parameter to be considered is the carbonyl index (CI). This is the most commonly used indicator for monitoring the chemical oxidation of polyolefins, and it is worth measuring their values in the just processed films, since its comparison provides an idea about the oxidation resistance of the materials under processing conditions. For that purpose, transmission spectra were used in order to track the oxidation level throughout the film thickness. The following Equation (5) was used for this calculation [23]:

(5) $$CI=\frac{Area\;down\;the\;band\;1850-1650\;{\mathrm{cm}}^{-1}}{Area\;down\;the\;band\;1500-1397\;{\mathrm{cm}}^{-1}}$$

The different carbonyl indexes are displayed in Table 6, where significant changes can be observed. In particular, the incorporation of norbornene into the polymeric chains seems to significantly decrease the CI. This apparent greater endurance against oxidation is discussed in the next section.

**Figure 4:**
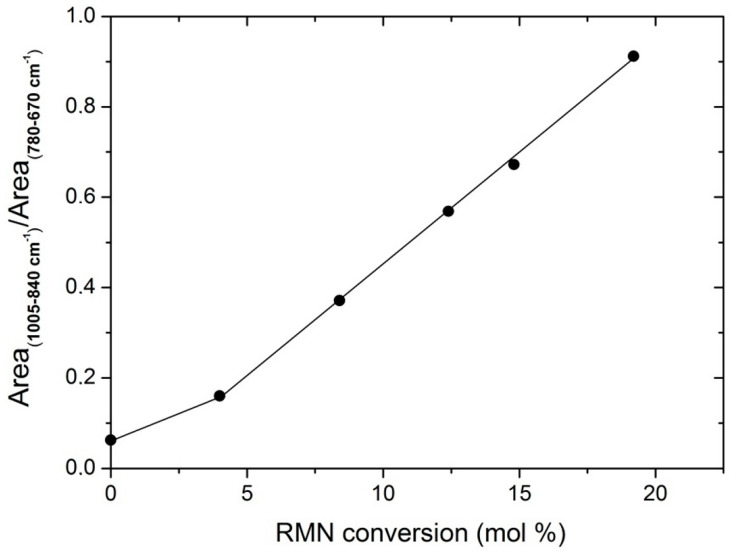
Calibration curve of the comonomer content estimated by FTIR and NMR from all the polymers.

Transmission spectra were also used for analyzing the composition of the prepared copolymers, using as a reference the data obtained by ^13^C RMN. As mentioned above, the area placed between 1005 and 840 cm^−1^ is associated with the vibrational deformation modes of norbornene, and has been normalized with respect to the peaks within the 780 and 670 cm^−1^ interval, which are related to polyethylene. Figure 4 shows that the data obtained by FTIR and NMR correlates rather well, fitting a straight line, except at very low contents.

### 3.3. Thermo-Oxidative Stability

**Figure 5:**
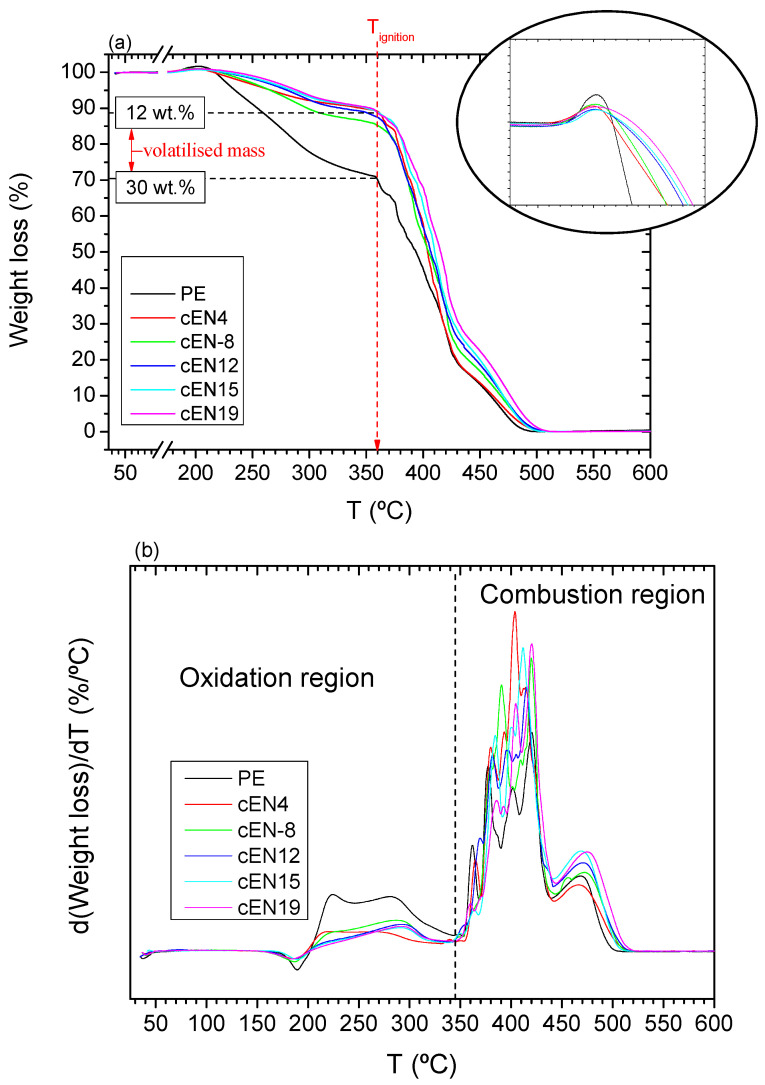
TGA curves (**a**) and DGTA curves (**b**) for all the samples.

The inspection of raw TGA curves, represented at 2 °C/min in Figure 5a, for the PE homopolymer and its copolymers, allows remarking that the weight loss is a two-step process, no matter the norbornene content. In all cases, the evolution of volatiles occurs gradually in the first stage up to a temperature of around 360 °C, from which it speeds up in a series of consecutive jumps, leading to the total volatilization of the material. These two stages can be clearly seen in the DGTA curves, as displayed in Figure 5b. While the latter stage is ascribed in literature to the ignition of PE-based material [24], the former corresponds to the oxidation, and allows the remarking of net differences between the PE and the copolymers, as it apparently seems to depend on the composition. The present study only addresses this process.

It is clear that the simple insertion of a low content of norbornene units (sample cEN4) causes the evolution of volatiles to be slowed down, and the fraction of the material involved in the oxidation to be significantly smaller than in the neat PE (see 12 wt% vs 30 wt%), as indicated in Figure 5a. Accordingly, the oxygen take-up preceding the weight loss is more noticeable in the homopolymer, as the mass fraction involved is larger (see magnification in Figure 5a). Such differences suggest a correlation of the behavior with the copolymer composition, with it being feasible that the reduced oxidation rate could be caused by the changes undergone within the material as a result of the norbornene insertion.

**Figure 6:**
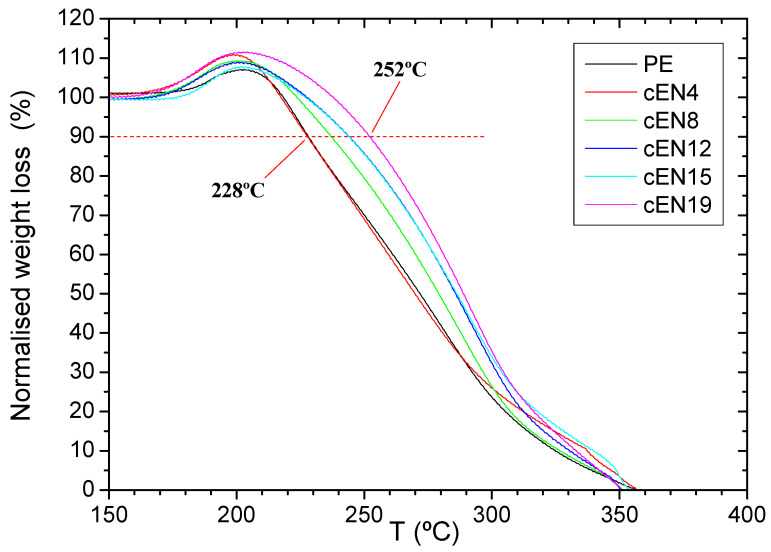
Normalized weight loss for the samples under oxidation conditions.

A more accurate and reliable picture about what is happening in oxidation is revealed when the weight loss is normalized at this stage, as shown in Figure 6. From this representation, it is first evident that the oxygen uptake is relatively higher for the copolymers than for the PE, and secondly, that the delay in weight loss is directly linked to the norbornene content. In fact, for a 10% weight loss, the temperature shift is 24 °C for cEN19 when comparing the 2 °C/min curves.

These remarks are consistent with both chemical and rheological changes experienced by the materials, as a consequence of the insertion of increasing norbornene contents into PE chains. On the one hand, norbornene units provide tertiary C-H bonds that are more susceptible to oxidation than main chain methylene groups, and can justify the relatively higher oxidation degree of copolymers, i.e., a higher hydroperoxide build-up, that is reached before the weight loss. On the other hand, however, changes in the rheology of the materials with the norbornene content may account for the lower mass fractions involved in copolymer oxidation. In particular, it is well-known that cyclo-olefins make intra-chain dynamics more difficult, thus being responsible for the observed increase of T_g_ [1,2,3], as shown in Table 1.

Moreover, it has been reported that the addition of cyclic counits into PE, i.e., COC copolymers, in the range of 5 to 15 vol.% increases the complex viscosity at 240 °C [25], which is evidence that intermolecular mobility is also hindered by the presence of norbornene, at least in the composition range analyzed in this study.

This motion hindrance is also observed in the solid state from the dependence of storage modulus at 20 °C, determined at 3 Hz in dynamic mechanical analysis in the specimens. Their respective values are 1105, 130, 75, 28, 60 and 1310 MPa for PE, EN4, EN8, EN12, EN15 and EN19, respectively. Two opposite effects are taking place. On one hand, PE is a crystalline polymer showing a high degree of crystallinity. Norbonene incorporation decreases its chain regularity, and accordingly, its crystallization capability. Thus, storage modulus is diminished until content in norbornene, which is a rigid comonomer, is high enough to overcome the loss of crystallinity. Then, the storage modulus starts to increase with composition, showing the EN19 specimen a value higher than that found in PE homopolymer. The former is completely amorphous, and crystallinity in the neat PE is about 60%.

Coming back to the molten state, slower intra- and intermolecular dynamics can delay both the access of oxygen and the diffusion of radical activity generated through the bulk of the material in the molten copolymers. However, it should be considered that intra-chain features may also contribute themselves to the deceleration of volatiles emissions. In fact, in the case of preferential oxidation of norbornene units, the process might occur without polymer chain scission, since breakage in a cyclic unit may not necessarily lead to a chain split. Thus, norbornene units would act as effective cleavage disruptors.

**Figure 7:**
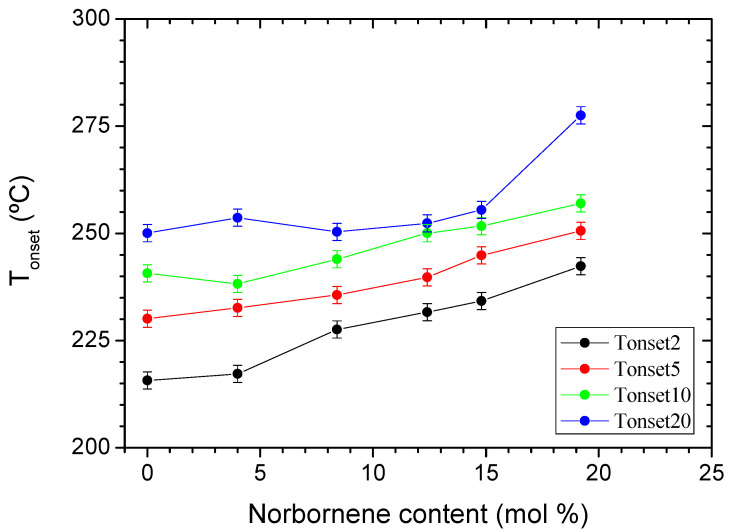
Onset and offset temperatures as a function of the norbornene contents (bars correspond to an error of ±2 °C).

Similarly, the slow molecular dynamics can act as a barrier to the release of volatiles, and this feature must not be discarded a priori as a factor contributing to the shifting of TGA curves towards higher temperatures. Whatever the mechanism, the actual fact is that norbornene units cause these COC copolymers to exhibit higher stability than the pristine PE, as can be deduced from Figure 7, which collects the variations of the onset temperatures of the weight loss as a function of the norbornene contents, obtained for the different heating rates.

**Figure 8:**
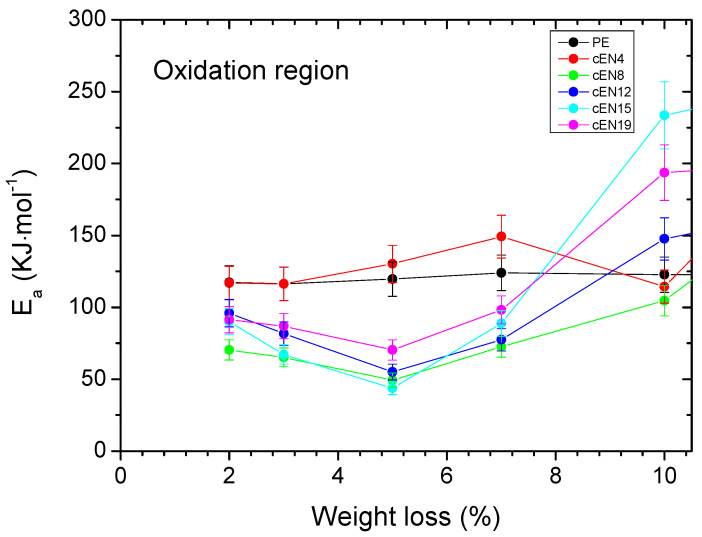
Activation energies associated with the weight loss (bars correspond to a relative error of 10%).

The calculus of the activation energy (E_a_) provides some support to the preferential involvement of tertiary CH bonds, i.e., norbornene units, in the oxidation stage. Figure 8 leaves no doubt about the decreasing of the apparent Ea value for COC copolymers in the oxidation stage. Although the measurement error does not allow a correlation to be made with the norbornene content, this shift points to the expected smaller intrinsic stability of cyclic co-units. Accordingly, the observed delay in volatiles releasing must be related to the influence of norbornene insertion on both inter- and intramolecular characteristics, particularly on chain dynamics and the possibility of oxidation without chain scission, respectively.

### 3.4. Thermal Stability under N_2_ Atmosphere

**Figure 9:**
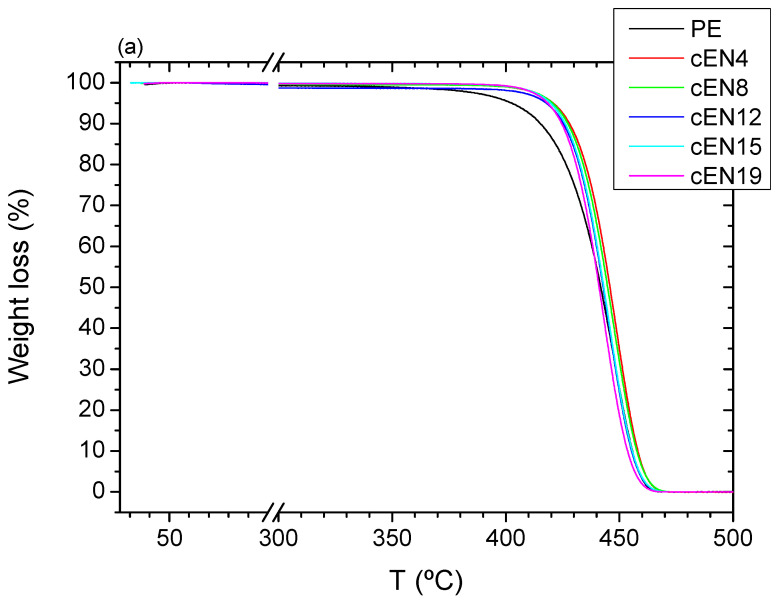

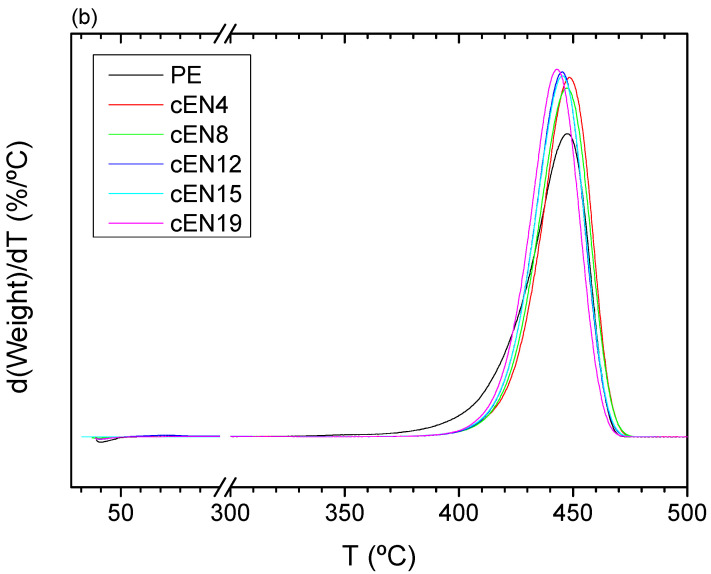
TGA curves (**a**) and DGTA curves (**b**) for all the samples under inert conditions.

**Figure 10:**
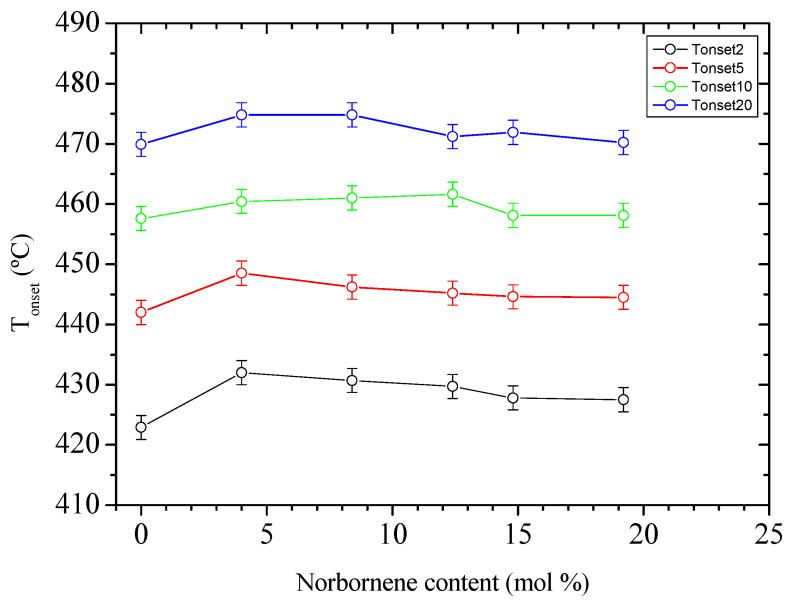
Onset temperatures of the degradation under nitrogen atmosphere (bars correspond to an error of ±2 °C).

Similar to the case of oxidation, the pyrolysis of EN copolymers takes place at temperatures higher than that of PE, despite the whole process taking place in a single step, as can be seen in Figure 9, which shows the TGA runs and their corresponding DTGA curves, respectively. However, the stabilization seems not to be proportional to the comonomer content in this case, since the starting temperature for the weight loss moves towards a higher value for sample EN-4, and hardly changes for further norbornene contents. Such variations are shown in Figure 10.

**Figure 11:**
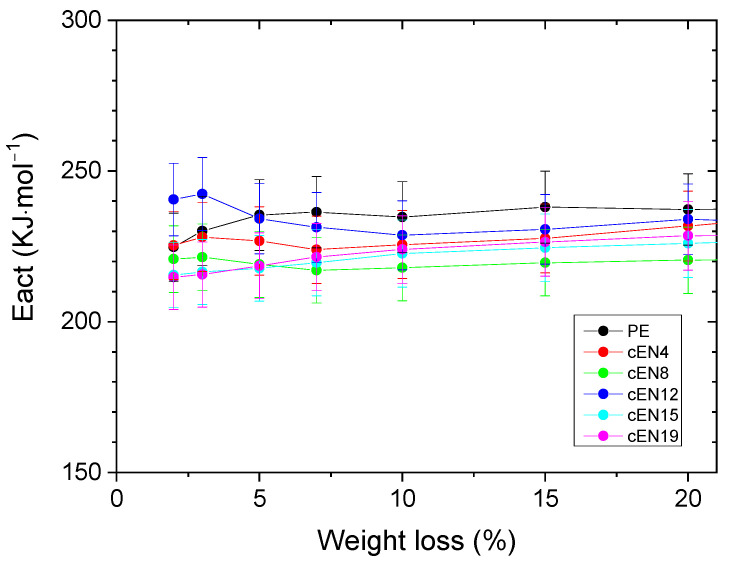
Activation energies associated with the weight loss under inert conditions (bars correspond to a relative error of 5%).

The lack of correlation between the Ti evolution and norbornene content allows for suspecting that changes in chain mobility, i.e., in viscosity, are likely the main cause for the observed delay in volatiles releasing, either because an enhanced barrier effect is playing or because chain scission is extremely slowed down, or both. In this respect, the influence of norbornene content on the Ea value may provide some insight. Figure 11 displays the variation of Ea with the pyrolysis progress for the different specimens. It is clear that the Ea required for the weight loss to start (up to 10 wt.%) is lower as the content in comonomer increases. This fact clearly suggests that norbornene units are mainly involved in the early stages of pyrolysis, and that main chain scission would preferentially occur at comonomer links. It is reasonable to assume that norbornene units act as weak points into chains, not only because they are overstressed bicycles, but because they are local stiffness points where mobility disruptions might concentrate thermal stress [12,26,27,28]. Therefore, the delay in volatiles releasing might be related to the above-mentioned enhanced barrier effect in EN copolymers, as well as the role of comonomers as both weak chain points and disruptors of the intra-chain scission mechanism, which is the main source of volatiles at the beginning of pyrolysis [29,30].

The behavior of EN copolymers, showing higher onset temperatures for the weight loss oxidation process than PE, but lower Ea for pyrolysis, has important consequences for both the applicability and sustainability of these materials. They are thus expected, on one hand, to exhibit higher performance against oxidation, while on the other hand, be converted into other chemicals at the end of their service life by using less energy-demanding processes, due to their lower Ea of pyrolysis.

## 4. Conclusions

A family of random COC copolymers based on ethylene and norbornene were synthesized in a broad range of compositions (up to 19 mol % in norbornene) through metallocene catalysis. A negative comonomer effect was observed over the catalytic activity in the kinetic analysis. Furthermore, the microstructure in composition shows a clear relationship with the comonomer ratio in the feeding, which can be described by a second-grade equation. The norbornene units incorporated in the polymeric chains are isolated, observing traces of dyads exclusively for the material with the highest content (19 mol %).

The thermal and thermo-oxidative stability, evaluated by thermogravimetric analysis, is higher for copolymers with norbornene than for the neat PE, which could be interesting for diverse applications where temperature can play an important role. On the other hand, their degradation, either under an inert or oxidizing atmosphere, exhibits an activation energy lower than those observed for PE. This is specifically interesting for chemical recycling, with the main finding being that the process would require lower energy consumption in the COC copolymers. This fact could be associated with the slowing effect that norbornene units have on the radical mechanisms that generate volatile species, and will be deeply addressed in future works.

## Figures and Tables

**Table 1:** Synthetic parameters involved in the synthetic procedure.

Sample	Initial Pressure (Bar)	Norbornene/Ethylene Molar Feeding Ratio	[Al]/[Zr]
PE	1.58	0	1083
cEN4	1.59	0.066	1083
cEN8	1.53	0.137	1083
cEN12	1.57	0.187	1083
cEN15	1.56	0.269	1083
cEN19	1.59	0.386	1064

**Table 2:** Conditions used for the processing of materials.

Sample	Processing Conditions	
	T (°C)	P (Bar)
PE	190	110
cEN4	160	110
cEN8	160	100
cEN12	150	50
cEN15	140	25
cEN19	170	160

**Table 3:** Yields, comonomer content and activity for the resultant materials.

Sample	Yield (g)	Comonomer Content * (mol %)	Activity (kg/h·mol Zr)
PE	0.84	0	685
cEN4	1.10	4.0	1051
cEN8	1.00	8.4	709
cEN12	1.32	12.4	315
cEN15	1.30	14.8	453
cEN19	1.54	19.2	636

* Norbornene content estimated by ^13^C RMN.

**Table 4:** Compositional sequences for resultant copolymers.

Compositional Sequence	Signal (ppm)	Carbon Nucleus
EEEEEEE	27.73	S_δ+δ+_
NEEEEE	27.79	S_δδ+_
NEEEE	27.92	S_βδ+_
NEEE	28.02	S_γδ_, S_γδ+_
NEEE	28.12	S_αδ+_
EENEE+NEEN	28.26	S_αδ_
NEEN	28.39	S_βγ_
EENENEE	28.53	S_αβ_
ENNE	29.68	C6

**Table 5:** Compositional triads and average length sequences of ethylene for all the prepared copolymers.

Sample	EEE	EEN	NEN	ENE	nE
cEN4	87.7	8.2	0	4.1	23
cEN8	73.0	18.0	0	9	10
cEN12	64.9	23.4	0	11.7	7
cEN15	55.7	26.8	2.1	15.5	5
cEN19	37.1	34.3	4.8	21.9	3

**Table 6:** Carbonyl index for all the samples.

Sample	CI
PE	0.0148
cEN4	0.0113
cEN8	0.0084
cEN12	0.0061
cEN15	0.0106
cEN19	0.0073

## Data Availability

Not applicable.
